# Supplementary material for: Introducing Polar Groups in Porous Aromatic Framework for Achieving High Capacity of Organic Molecules and Enhanced Self-Cleaning Applications
Source: Molecules. 2022 Sep 19;27(18):6113. doi: 10.3390/molecules27186113 (PMC9501117; doi:10.3390/molecules27186113)
Supplement: Supplementary file 1 [file molecules-27-06113-s001.zip › molecules-1914784-supplementary.pdf]

molecules

Supplementary Materials

Introducing polar groups in porous aromatic framework for achieving high capacity of organic molecules and enhanced self-cleaning applications

Zhuojun Yan[1], Yimin Qiao[1], Qiqi Sun[2], Bo Cui[1], Bin Feng[1], Naishun Bu[2], Kuo Chu[2*], Xianghui Ruan[3], Ye Yuan[3], Yajie Yang[3*] and Lixin Xia[1,4*]

[1] College of Chemistry, Liaoning University, Shenyang 110036, China
[2] School of Environmental Science, Liaoning University, Shenyang 110036, China
[3] Key Laboratory of Polyoxometalate and Reticular Material Chemistry of Ministry of Education, Northeast Normal University, Changchun 130024, China
[4] Yingkou Institute of Technology, Yingkou 115014, China
* Correspondence: chukuolnu@163.com (K.C.); yangyj557@nenu.edu.cn (Y.J.Y.); lixinxia@lnu.edu.cn (L.X.X.)

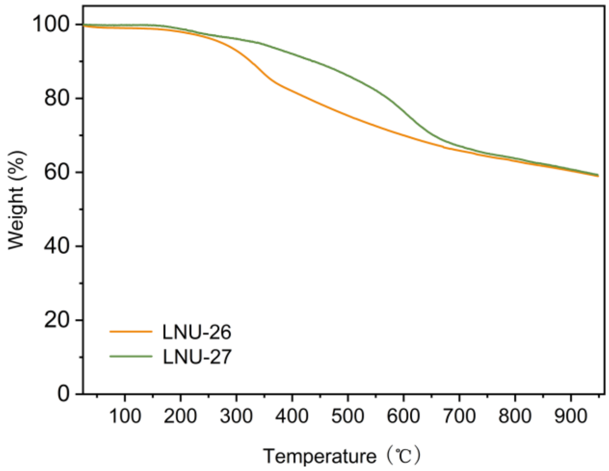

Figure S1 TGA curves for PAF solids under nitrogen atmosphere.

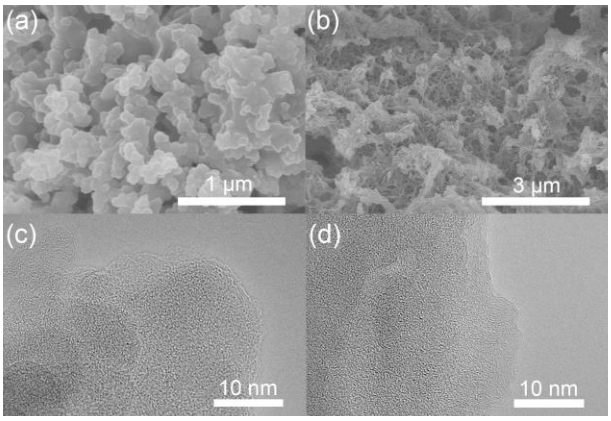

Figure S2 SEM images for (**a**) LNU-26 and (**b**) LNU-27; TEM images for (**c**) LNU-26 and (**d**) LNU-27.

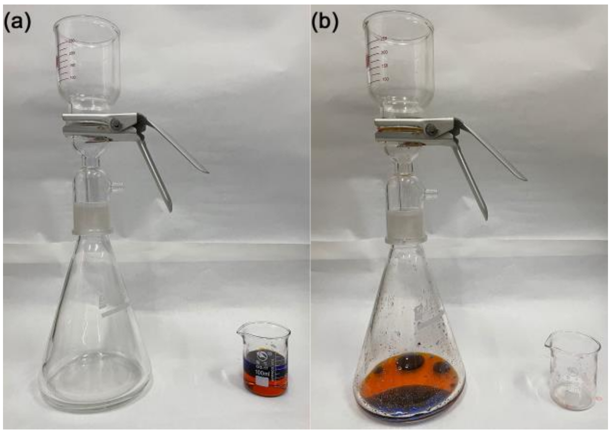

Figure S3 Photographs of the separation of chloroform (dyed with methyl red) and water (dyed with methyl blue) using the raw polyester fabric.
